# Impact of stroke imaging selection modality on endovascular thrombectomy outcomes in the early and extended time windows: A meta‐analysis

**DOI:** 10.1002/brb3.3530

**Published:** 2024-08-01

**Authors:** Jiwei Wu, Zhi Peng, Hengzhu Zhang

**Affiliations:** ^1^ Department of Neurosurgery Northern Jiangsu People's Hospital Affiliated to Yangzhou University Yangzhou Jiangsu China

**Keywords:** ischemic stroke, meta‐analysis, neuroimaging, perfusion imaging, thrombectomy, treatment outcome

## Abstract

**Background:**

The effect of imaging selection modality on endovascular thrombectomy (EVT) clinical outcomes in patients with acute ischemic stroke due to large vessel occlusion (AIS‐LVO) remains unclear. This study aims to compare post‐EVT outcomes in patients with AIS‐LVO who underwent basic imaging (computed tomography with or without computed tomography angiography) and advanced imaging (computed tomography perfusion or magnetic resonance imaging) in early and late time windows.

**Method:**

A systematic literature search was conducted on PubMed, Cochrane Library, and Embase databases from inception until June 10, 2023. Studies investigating the relationship between the imaging selection modality and post‐EVT outcomes in patients with AIS‐LVO were retrieved. A random‐effects model was used to pool the effect estimates of successful reperfusion, symptomatic intracranial hemorrhage (sICH), functional independence, and mortality. The meta‐analysis was performed using Review Manager software v.4.3, and the outcomes were assessed using odds ratios (ORs) and 95% confidence intervals (CIs).

**Result:**

A total of 13 non‐randomized observational studies, comprising 19,694 patients, were included in this meta‐analysis. In the early time windows, AIS‐LVO patients receiving advanced imaging demonstrated a higher likelihood of functional independence (OR, 1.25, 95% CI, 1.08–1.46) and a lower risk of mortality (OR,.73 95% CI,.61–.86) compared to those receiving basic imaging. In the extended time windows, AIS‐LVO patients undergoing advanced imaging had a lower mortality rate (OR,.79, 95% CI,.68–.92). Regardless of the time of onset, there were no significant differences between the two groups in terms of sICH or successful reperfusion.

**Conclusion:**

Advanced imaging combined with EVT may achieve better clinical outcomes in patients with AIS‐LVO. Further high‐quality studies are needed to validate these findings.

## Graphical abstract

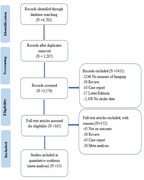

## 1 INTRODUCTION

Multiple randomized controlled trials published in 2015 (Berkhemer et al., 2015; Campbell et al., 2015; Goyal et al., 2015; Jovin et al., 2015; Saver et al., 2015) demonstrated that endovascular thrombectomy (EVT) brought significant clinical benefits to patients with acute ischemic stroke due to large vessel occlusion (AIS‐LVO) within 6 h of onset (Goyal et al., 2016). Since then, endovascular therapy has emerged as an important treatment method for AIS‐LVO patients. Subsequent trials such as DAWN (Nogueira et al., 2018) and DEFUSE 3 (Albers et al., 2018) further underscored the efficacy of EVT in AIS‐LVO patients carefully selected through advanced neuroimaging modalities like magnetic resonance imaging (MRI) or computed tomography perfusion (CTP). These findings have taken endovascular therapy from the time window into the era of tissue windows.

According to the 2019 Guidelines for Mechanical Thrombectomy in Acute Ischemic Stroke (Turc et al., 2019), adult patients with AIS are selected for EVT without the need for advanced imaging in the early time window (defined as 0–6 h from the last known time) (evidence level: Level 2 recommendation, Level B evidence). However, for patients with advanced time windows (defined as 6–24 h from the last known time), advanced imaging selection is necessary (with moderate quality of evidence and strong recommendations). Emergency MRI or CTP is uncommon in low‐grade stroke centers (Wintermark et al., 2015). Some studies suggest that advanced imaging techniques may inadvertently exclude patients who could have potentially benefited from endovascular therapy (García‐Tornel et al., 2021). Therefore, many centers predominantly rely on basic imaging techniques, such as non‐contrast computed tomography (NCCT), to calculate the Alberta Stroke Program Early CT Score (ASPECTS) for patient selection in endovascular treatment (Nguyen et al., 2022). However, the utilization of advanced imaging modalities can lead to the exclusion of patients with ineffective reperfusion, thereby potentially enhancing clinical outcomes (Dhillon et al., 2023), and there is a growing trend in advanced stroke centers to apply advanced imaging within early time windows.

The controversy surrounding the optimal imaging approach for EVT in patients with AIS‐LVO presents treatment challenges for clinical practitioners. Although previous studies (Dong et al., 2023; Kobeissi et al., 2023) have attempted to address this issue, their conclusions remain inconsistent. In addition, they did not conduct subgroup analysis based on time windows. Therefore, current research fails to offer definitive guidance on this issue. Therefore, this study represents the first comprehensive analysis to explore the impact of advanced selective imaging on post‐EVT outcomes in the early and extended time windows.

## 2 METHOD

This study was conducted according to the Preferred Reporting Items for Systematic Reviews and Meta‐Analyses (PRISMA) statement (Moher et al., 2009) (Supplementary Material 1). The study protocol (INPLASY202370026) has been registered on the International Platform of Registered Systematic Review and Meta‐Analysis Protocols (INPLASY, https://inplasy.com/).

### 2.1 Literature retrieval and search strategy

We conducted a systematic search of PubMed, Embase, and the Cochrane Library for relevant studies on imaging for EVT in patients with AIS‐LVO, without language restrictions, from inception until June 10, 2023. A combination of keywords and/or medical subject headings terms were used, including “ischemic stroke,” “LVO,” “CTP,” “MRI,” “CT angiography,” “NCCT,” and “mechanical thrombectomy.” The detailed search strategy is provided in Supplementary Materials 2.

### 2.2 Eligibility criteria

Studies that met the specified PICO criteria (Patient, Intervention, Comparison, and Outcome) were included in the analysis.

Patients: Individuals with AIS‐LVO.

Intervention: AIS‐LVO patients treated with mechanical thrombectomy.

Comparison: Advanced imaging (MRI and/or CTP) versus basic imaging (CT and/or CT angiography).

Outcome: Outcome variables included functional independence (defined as an mRS score of 0–2), mortality, successful reperfusion (defined as a Thrombolysis in Cerebral Infarction [TICI] score of 2b‐3), and spontaneous intracerebral hemorrhage (sICH).

Studies that did not meet the inclusion criteria, such as case reports, reviews, letters, and meta‐analyses, were excluded from the analysis.

### 2.3 Data extraction

Two investigators conducted an independent review of titles and abstracts to assess the possible relevance of the studies. Any disagreements were resolved through consensus or discussion with a third investigator. The extracted information from the selected studies included the first author's name, publication year, setting, study design, sample size, average age, symptom onset, NIHSS score, and primary endpoints. Additionally, raw data for each study was recorded.

### 2.4 Assessment of risk of bias

All studies were assessed for the risk of bias by two independent investigators using the Newcastle–Ottawa scale (Stang, 2010). The studies were scored based on selection, comparability, and outcomes, with higher scores indicating higher study qualities. Generally, a Newcastle–Ottawa scale score of 7 or above is considered high quality. Any discrepancies between the investigators were resolved through discussion, with the occasional involvement of a third investigator as an arbitrator.

### 2.5 Statistical analysis

Patients with AIS‐LVO were stratified according to baseline imaging modality (basic vs. advanced) and treatment time window (0–6 h vs. 6–24 h from the last known well to puncture). Effect sizes were determined using odds ratios (ORs) and standardized mean differences along with their corresponding 95% confidence intervals (CIs). A random‐effect model that considers heterogeneity within and between studies while assuming that true effect sizes vary across studies was used for meta‐analysis. Heterogeneity was assessed using the *I* ^2^ index, with values exceeding 50% considered significant (Cumpston et al., 2019). Funnel plots were used to assess publication bias. Statistical analyses were conducted using the Review Manager software v.4.3.

## 3 RESULTS

### 3.1 Study characteristics

**FIGURE 1:**
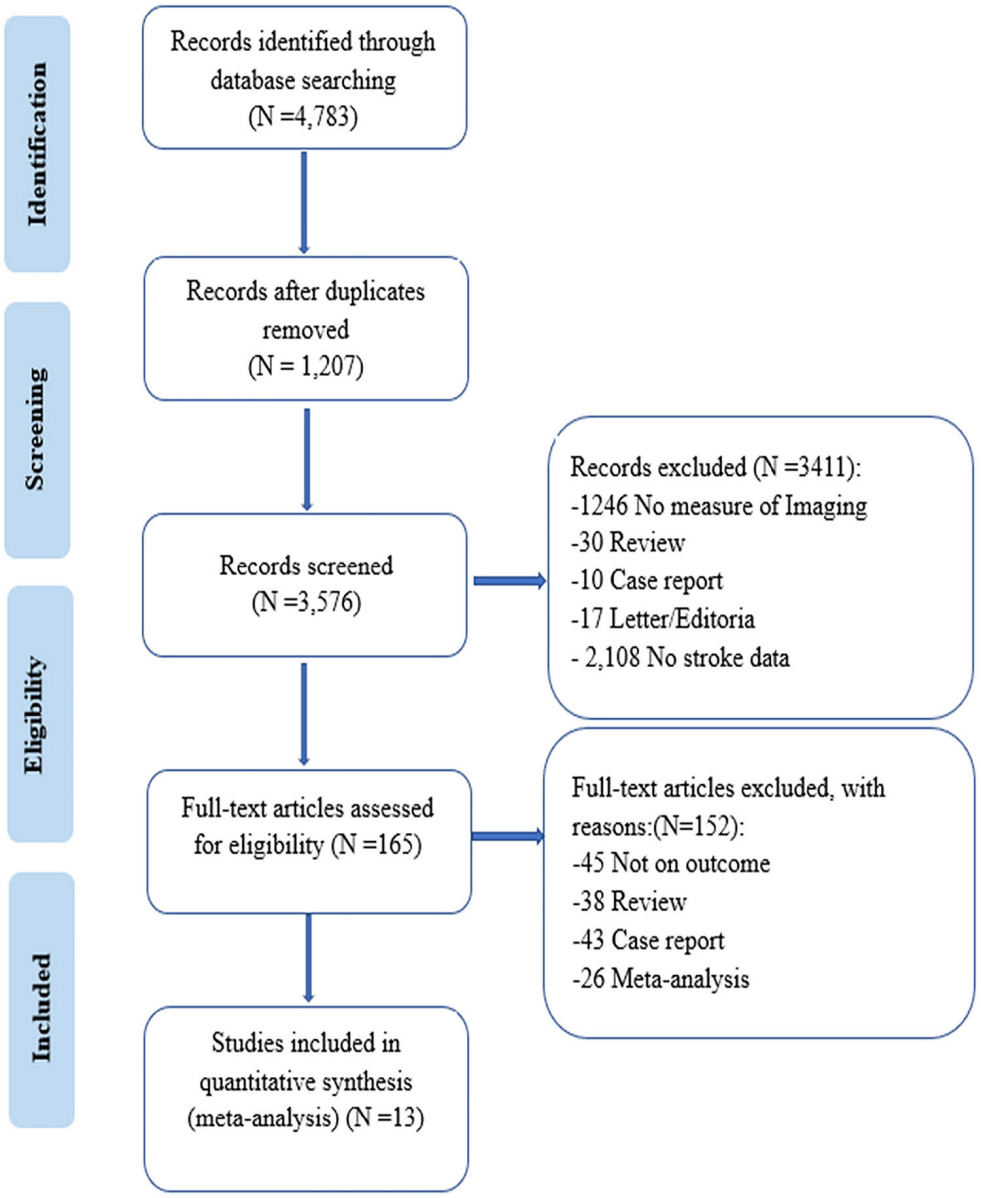
Flowchart of literature search and study selection.

The initial search yielded 4783 studies. Following the removal of duplicates and screening of titles and abstracts, 165 studies were subjected to full‐text review. To avoid heterogeneity in outcome estimates, 14 studies (Bouslama et al., 2017; Chalouhi et al., 2013; Dekker et al., 2021; Fischer et al., 2022; Garcia‐Esperon et al., 2023; Kim & Kim, 2020; Koneru et al., 2018; Krebs et al., 2022; Leslie et al., 2016; Lin et al., 2020; Prabhakaran et al., 2014; Provost et al., 2019; Sheth et al., 2013; Vagal et al., 2016) that compared the outcomes of patients who received EVT with the use of advanced imaging versus basic imaging were excluded from the meta‐analysis due to variability in treatment time windows. Furthermore, a study (Kim et al., 2019) that compared the results of diagnosis using advanced imaging versus basic imaging in patients with AIS‐LVO undergoing endovascular therapy, including arterial thrombolysis, was also excluded. Finally, 13 studies were ultimately included in the meta‐analysis (Almekhlafi et al., 2022; Cheng et al., 2023; Desai et al., 2019; Dhillon et al., 2022; Herzberg et al., 2021; Jadhav et al., 2022; Leslie et al., 2016; Miao et al., 2023; Nguyen et al., 2022; Nogueira et al., 2021; Porto et al., 2022; Sakakibara et al., 2023; Sarraj et al., 2018) (Figure 1). Among these studies, 6 compared outcomes within the early time window (0–6 h) (Dhillon et al., 2022; Jadhav et al., 2022; Leslie et al., 2016; Miao et al., 2023; Nogueira et al., 2021; Sakakibara et al., 2023), whereas 10 compared outcomes within the extended time window (6–24 h) (Almekhlafi et al., 2022; Cheng et al., 2023; Desai et al., 2019; Dhillon et al., 2022; Herzberg et al., 2021; Miao et al., 2023; Nguyen et al., 2022; Nogueira et al., 2021; Porto et al., 2022; Sarraj et al., 2018). This study included 13 trials with a total of 19,694 patients, of whom 8098 were assigned to the basic imaging group and 11,596 to the advanced imaging group. The patient characteristics are presented in Table 1. The excluded literature can be found in Supplementary Material 3.

**TABLE 1:** Characteristics of studies included in the systematic review and meta‐analysis.

			Sample size		Mean age (years)		Median NIHSS score			
Study ID	Setting	Study design	CTP or MRI	NCCT/CTA	CTP or MRI	NCCT/CTA	CTP or MRI	NCCT/CTA	Symptom onset	Outcome
Leslie (2016)	Single‐center	P	72	31	66 (53.5–72.5)	67 (61.3–81.8)	16.5 (3.4)	16.9 (4.7)	<6 h	②③⑤⑥
Sarraj (2018)	Multicenter	P	474	75	67	69	NR	NR	6–24 h	②
Desai (2019)	Multicenter	R	39	46	66	74	13	13	6–24 h	②⑥
Nogueira (2021)	Multicenter	P	553	399	65.8 ± 15.1	66.7 ± 14.5	16 (12–20)	15 (11–20)	<6 h, 6–24 h	②③④⑤
Herzberg (2021)	Multicenter	R	79	129	72.85 (11.56)	72.29 (12.66)	16.9 (2.4)	16.7 (3.4)	6–24 h	②③④⑤
Dhillon (2022)	Multicenter	P	917	3278	NR	NR	NR	NR	<6 h, 6–24 h	①②③④⑤
Porto (2022)	Multicenter	R	314	419	70 (60–81)	68 (57–78)	14 (8–19)	15 (10–19)	6–24 h	②③④⑤
Jadhav (2022)	Multicenter	P	610	738	67 (58–76)	68 (58–77)	17 (14–21)	17 (13–20)	<6 h	①②③④
Nguyen (2022)	Multicenter	P	1070	534	71.5 (61–80)	71 (58–81)	16 (11–19)	17 (13–21)	6–24 h	②③④
Almekhlafi (2022)	Multicenter	P	379	229	66.5 (14.9)	68.9 (14)	16 (10)	15 (11)	6–24 h	①②③④⑤
Cheng (2023)	Multicenter	R	102	102	66 (54–73)	63 (55–70)	15 (11–18)	14 (12–19)	6–24 h	①②③④
Miao (2023)	Multicenter	P	489	639	68 (59–76)	68 (56–74)	15 (11–19)	16 (13–20)	<6 h, 6–24 h	①②③④
Sakakibara (2023)	Multicenter	P	40	53	76.5 ± 10.2	76.5 ± 10.2	22 (18–26)	22 (17–26)	<6 h	①②③④

*Note*: Outcome: ①mRS 0‐1; ②mRS 0‐2; ③Mortality; ④sICH: spontaneous intracerebral hemorrhage; ⑤TICI 2b‐3; ⑥SH: Symptomatic hemorrhage.

Abbreviations: CTP, computed tomography perfusion; MRI, magnetic resonance imaging; NCCT, non‐contrast computed tomography; NR, no report; P, prospectively; R, retrospective.

### 3.2 Study quality and publication bias

The included studies were assessed using the Newcastle–Ottawa scale, and all studies were scored between 7 and 9 points, indicating a high level of quality and reliability in both methods and results. Further details regarding this assessment are provided in Supplementary Material 4. Funnel plot analysis revealed no evidence of study asymmetry in the reporting of unadjusted outcomes (Supplementary Material 5).

### 3.3 Early time windows for clinical outcomes

A pooled analysis of all patients as a single group demonstrated a significant difference in functional independence between the advanced imaging (817 of 1824, 44.79%) and the basic imaging (1568 of 4229, 37.1%; OR, 1.25, 95% CI, 1.08–1.46; Figure 2a) groups. No significant heterogeneity was observed (*I* ^2^ = 25%).

FIGURE 2Forest plot was compared between the advanced imaging group and the basic imaging group. (a, functional independence; b, sICH; c, successful reperfusion; d, mortality).

**Figure:**
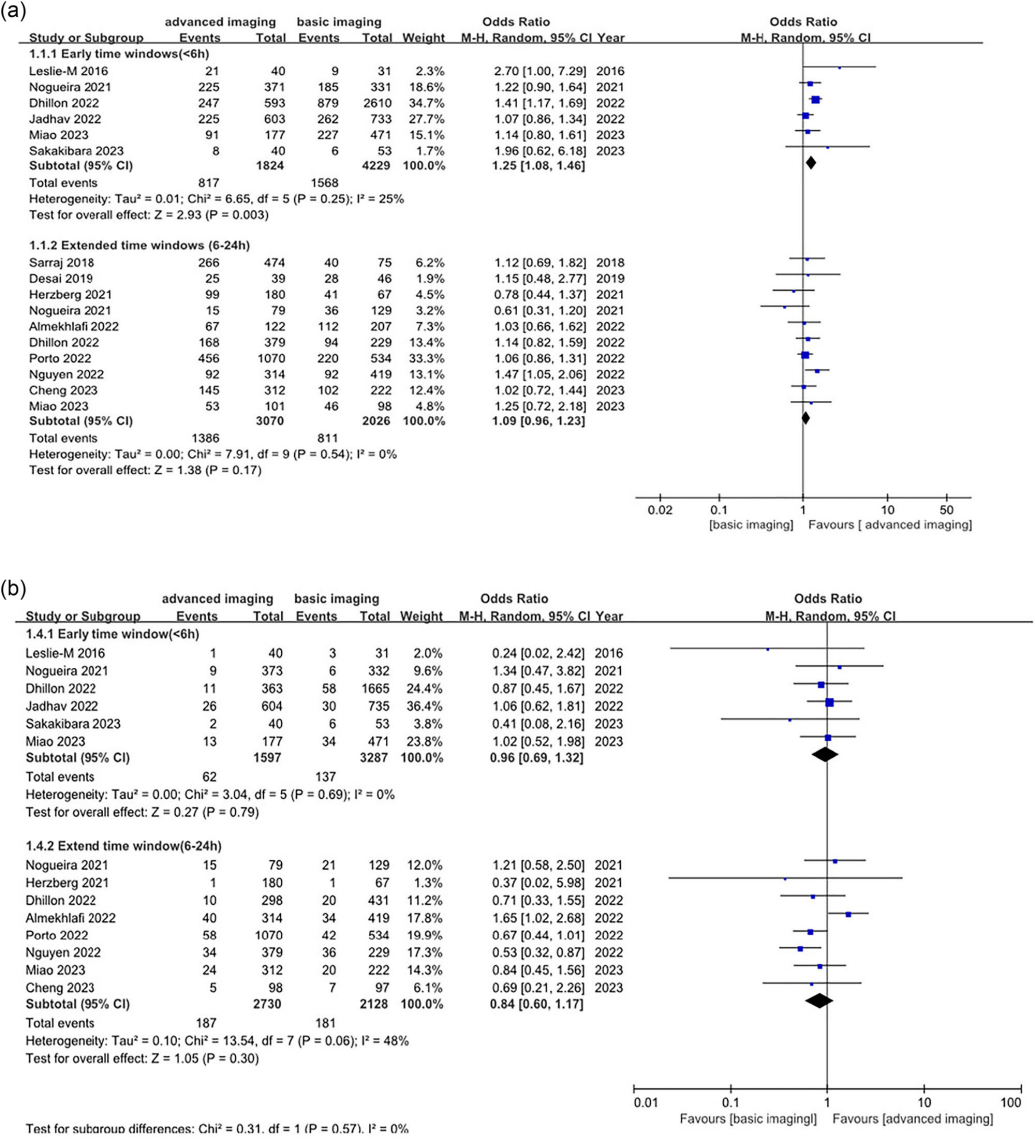

**Figure:**
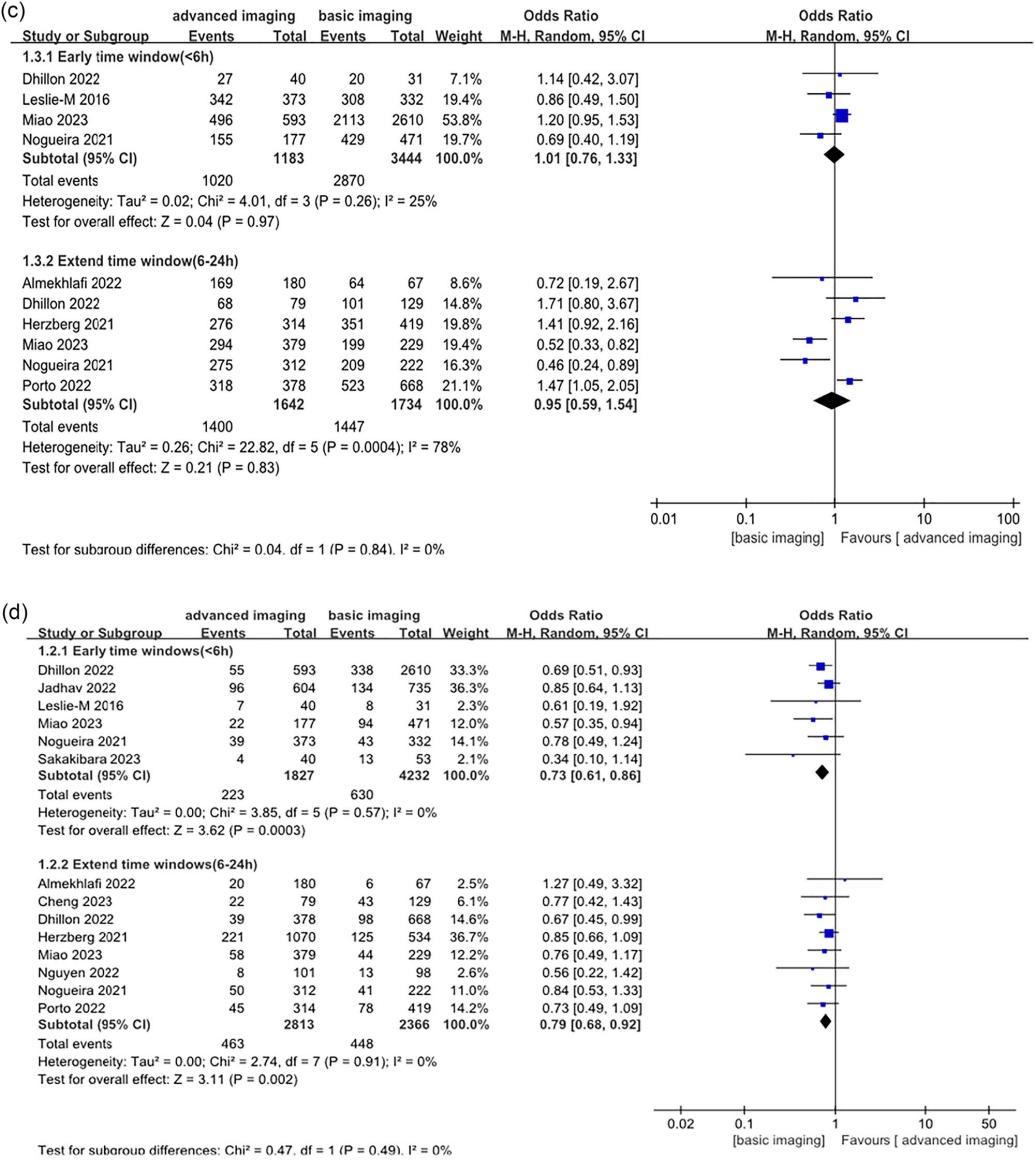

In the advanced imaging group, a slightly lower but statistically insignificant rate of sICH (62 of 1597, 3.88%) was noted compared to the basic imaging group (137 of 3287, 4.16%; OR,.96, 95% CI,.69–1.32; Figure 2b). There was no significant heterogeneity (*I* ^2^ = 0%).

There was no significant difference in successful reperfusion between the advanced imaging (1020 of 1183, 85.6%) and the basic imaging (2870 of 3444, 83.3%; OR, 1.01, 95% CI,.76–1.33; Figure 2c) groups. The heterogeneity was not significant (*I* ^2^ = 25%).

The advanced imaging group reported a statistically significantly lower mortality rate (223 of 1827, 12.2%) than the basic imaging group (630 of 4232, 14.8%; OR,.73, 95% CI,.61–.86; Figure 2d). No heterogeneity was found (*I* ^2^ = 0%).

### 3.4 Extended time windows for clinical outcomes

Functional independence was achieved in 1386 out of 3070 (45.1%) patients in the advanced imaging group, compared with 811 out of 2026 (40%) in the basic imaging group (OR, 1.09, 95% CI,.96–1.23; Figure 2a). There was no observed heterogeneity (*I* ^2^ = 0%).

The advanced imaging group reported a significantly lower rate of sICH (187 out of 2730, 6.85%) than the basic imaging group (181 out of 2128, 8.5%; OR,.84, 95% CI,.60–1.17; Figure 2b). There was no significant heterogeneity observed (*I* ^2^ = 48%).

There was no significant improvement in successful reperfusion rates between the advanced and basic imaging groups (85.2% vs. 83.4%; OR,.95, 95% CI,.59–1.54; Figure 2c), and considerable heterogeneity was present (*I* ^2^ = 78%).

Compared to baseline imaging, advanced imaging had lower mortality rates (16.4% vs. 14.7%; OR,.79, 95% CI,.68–.92; Figure 2d). No heterogeneity was observed (*I* ^2^ = 0%).

## 4 DISCUSSION

Our meta‐analysis revealed that the use of advanced imaging techniques was associated with higher functional independence and lower mortality in the early time window. However, in the extended time window, patients undergoing advanced imaging demonstrated a lower mortality rate. It is worth noting that there was no significant difference in the success rate of reperfusion and sICH between the two imaging methods at different time windows. Our results suggest that the advanced imaging techniques for EVT in the early and extended time windows may be effective.

A previous indirect meta‐analysis (Tsivgoulis et al., 2018) indicated that using advanced neuroimaging in AIS patients undergoing EVT was associated with increased functional independence. There were no significant differences in mortality and sICH rates between patients who underwent advanced neuroimaging and those who received basic neuroimaging. However, patients undergoing advanced imaging techniques typically received treatment within the 6–24 h time window, whereas those utilizing basic imaging techniques received treatment within the 0–6 h time window. This difference may result in variations in clinical characteristics and treatment strategies among the patients, prompting inquiries into the reliability of these research findings. One recent study has shown that the use of CTP imaging technology in the late time window is associated with higher success rates of reperfusion and lower mortality rates (Kobeissi et al., 2023). Based on previous research, we reasonably divided the patient time window and obtained similar results. Compared with previous studies, our study has significant advantages in sample size, imaging technology, and time window. Therefore, our conclusions are more convincing and applicable, providing important guidance for the treatment of AIS patients.

As is well known, TICI 2C or 3 reperfusion is associated with higher functional independence compared to TICI 2B reperfusion (Kaesmacher et al., 2018). Our meta‐analysis revealed no enhancement in reperfusion success but indicated higher functional independence. We suggest that besides assessing successful reperfusion based on TICI grading, basic imaging inadequately delineates the extent of ischemic injury and often fails to identify large infarctions. Complete reperfusion may not be beneficial and could even be detrimental in cases of significant irreversible tissue damage. Advanced imaging modalities offer a high level of accuracy in infarction, detection, and quantification, enabling the exclusion of EVT for patients with complete infarction. This highlights how advanced imaging techniques contribute to better functional outcomes (Campbell et al., 2015). Patients who underwent advanced imaging in the late time window exhibited a similar improving trend in functional prognosis (45.1% vs. 40%) but did not reach a statistically significant effect size. This could be due to the low proportion of late‐stage patients showing good prognosis. Further studies with larger sample sizes are warranted for verification.

Interestingly, our results demonstrated that using advanced imaging methods resulted in higher functional independence in the early time window as well as lower mortality rates in the early and late time windows. The recent TWIST Trial (Roaldsen Melinda et al., 2023) indicated that intravenous injection of Tenaperase did not show any benefits compared to non‐thrombolysis in awakening stroke patients (including AIS‐LVO) who underwent NCCT triage. Patients who receive CTP may have more favorable collateral blood flow, potentially contributing to their lower post‐EVT mortality rate. A recent study involving a large cohort confirmed a significant association between NCCT and increased mortality (Kobeissi et al., 2023). The reason may be due to incomplete manifestations of ischemia within 24 hours (Goyal et al., 2020). However, due to the fallacy of denominators (Goyal, 2014), the results of this study should be interpreted with caution when considering the paradigm of optical imaging selection. For example, the benefits of improving functional outcomes by selection may have more favorable target mismatch characteristics in patients, whereas excluding patients with broader tissue characteristics may still benefit from EVT treatment efficacy (Lopez‐Rivera et al., 2020).

This study has several limitations. First, the potential effects of confounding variables cannot be fully controlled, which may lead to result bias. Furthermore, it is not possible to directly compare patients who meet baseline imaging standards but not advanced imaging standards regarding their eligibility for EVT and drug therapy. In addition, the lack of advanced imaging screening standards may affect the variability of outcome measurements. Finally, most of the included studies are retrospective in nature, underscoring the need for validation through higher quality prospective and randomized studies.

## 5 CONCLUSION

We demonstrate that the use of advanced imaging techniques can better identify patients who are eligible for EVT, leading to improved outcomes. Our findings may provide valuable insights for stroke physicians, patients, and caregivers, helping them understand the potential benefits of advanced imaging examinations. Further high‐quality clinical trials are required for validation.

## AUTHOR CONTRIBUTIONS

**Jiwei Wu**: Conceptualization; methodology; software; data curation; investigation; validation; formal analysis; supervision; writing—original draft. **Zhi Peng**: Funding acquisition; visualization. **Hengzhu Zhang**: Project administration; resources; writing—review and editing.

## CONFLICT OF INTEREST STATEMENT

The authors declare that there are no conflicts of interest regarding the publication of this article.

## FUNDING INFORMATION

No funding was received for the study.

### PEER REVIEW

The peer review history for this article is available at https://publons.com/publon/10.1002/brb3.3530

## Supporting information

Supplementary Materials

## Data Availability

All data generated or analyzed during this study are included in this article and/or its Supplementary Material files. Further inquiries can be directed to the corresponding author.
