# Supplementary material for: Impact of stroke imaging selection modality on endovascular thrombectomy outcomes in the early and extended time windows: A meta‐analysis
Source: Brain Behav. 2024 Aug 1;14(8):e3530. doi: 10.1002/brb3.3530 (PMC11293622; doi:10.1002/brb3.3530)

Supplementary Materials 1

**
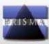
**

PRISMA 2020 Checklist

| **Section and Topic** | **Item#** | **Checklist item** | **where item**  **is reported** |  |
| --- | --- | --- | --- | --- |
| **TITLE** | | | |  |
| Title | 1 | Identify the report as a systematic review. | Page1 |  |
| **ABSTRACT** | | | |  |
| Abstract | 2 | See the PRISMA 2020 for Abstracts checklist. | Page1-2 |  |
| **INTRODUCTION** | | | |  |
| Rationale | 3 | Describe the rationale for the review in the context of existing knowledge. | Page2-3 |  |
| Objectives | 4 | Provide an explicit statement of the objective(s) or question(s) the review addresses. | Page2-3 |  |
| **METHODS** | | | |  |
| Eligibility criteria | 5 | Specify the inclusion and exclusion criteria for the review and how studies were grouped for the syntheses. | Page3-4 |  |
| Information sources | 6 | Specify all databases, registers, websites, organisations, reference lists and other sources searched or consulted to identify studies. Specify the date when each source was last searched or consulted. | Page3 |  |
| Search strategy | 7 | Present the full search strategies for all databases, registers and websites, including any filters and limits used. | Page3 |  |
| Selection process | 8 | Specify the methods used to decide whether a study met the inclusion criteria of the review, including how many reviewers screened each record and each report retrieved, whether they worked independently, and if applicable, details of automation tools used in the process. | Page4 |  |
| Data collection process | 9 | Specify the methods used to collect data from reports, including how many reviewers collected data from each report, whether they worked independently, any processes for obtaining or confirming data from study investigators, and if applicable, details of automation tools used in the process. | Page4 |  |
| Data items | 10a | List and define all outcomes for which data were sought. Specify whether all results that were compatible with each outcome domain in each study were sought (e.g. for all measures, time points, analyses), and if not, the methods used to decide which results to collect. | Page3-4 |  |
|  | 10b | List and define all other variables for which data were sought (e.g. participant and intervention characteristics, funding sources). Describe any assumptions made about any missing or unclear information. | Page3-4 |  |
| Study risk of bias  assessment | 11 | Specify the methods used to assess risk of bias in the included studies, including details of the tool(s) used, how many reviewers assessed each study and whether they worked independently, and if applicable, details of automation tools used in the process. | Page4 |  |
| Effect measures | 12 | Specify for each outcome the effect measure(s) (e.g. risk ratio, mean difference) used in the synthesis or presentation of results. | Page4-5 |  |
| Synthesis methods | 13a | Describe the processes used to decide which studies were eligible for each synthesis (e.g. tabulating the study intervention characteristics and comparing against the planned groups for each synthesis (item #5)). | Page4 |  |
|  | 13b | Describe any methods required to prepare the data for presentation or synthesis, such as handling of missing summary statistics, or data conversions. | Page4-5 |  |
|  | 13c | Describe any methods used to tabulate or visually display results of individual studies and syntheses. | Page4-5 |  |
|  | 13d | Describe any methods used to synthesize results and provide a rationale for the choice(s). If meta-analysis was performed, describe the model(s), method(s) to identify the presence and extent of statistical heterogeneity, and software package(s) used. | Page4-5 |  |
|  | 13e | Describe any methods used to explore possible causes of heterogeneity among study results (e.g. subgroup analysis, meta-regression). | Not applicable |  |
|  | 13f | Describe any sensitivity analyses conducted to assess robustness of the synthesized results. | Page5 |  |
| Reporting bias  assessment | 14 | Describe any methods used to assess risk of bias due to missing results in a synthesis (arising from reporting biases). | Page5 |  |
| Certainty assessment | 15 | Describe any methods used to assess certainty (or confidence) in the body of evidence for an outcome. | Page5 |  |
| **RESULTS** | | | |  |
| Study selection | 16a | Describe the results of the search and selection process, from the number of records identified in the search to the number of studies included in the review, ideally using a flow diagram. | Page5 |  |
|  | 16b | Cite studies that might appear to meet the inclusion criteria, but which were excluded, and explain why they were excluded. | Page5 |  |
| Study characteristics | 17 | Cite each included study and present its characteristics. | Page5 |  |
| Risk of bias in studies | 18 | Present assessments of risk of bias for each included study. | Page5 |  |
| Results of individual  studies | 19 | For all outcomes, present, for each study: (a) summary statistics for each group (where appropriate) and (b) an effect estimate and its precision (e.g. confidence/credible interval), ideally using structured tables or plots. | Page6 |  |
| Results of syntheses | 20a | For each synthesis, briefly summarise the characteristics and risk of bias among contributing studies. | Page6 |  |
|  | 20b | Present results of all statistical syntheses conducted. If meta-analysis was done, present for each the summary estimate and its precision (e.g. confidence/credible interval) and measures of statistical heterogeneity. If comparing groups, describe the direction of the effect. | Page6 |  |
|  | 20c | Present results of all investigations of possible causes of heterogeneity among study results. | Page6 |  |
|  | 20d | Present results of all sensitivity analyses conducted to assess the robustness of the synthesized results. | Page6 |  |
| Reporting biases | 21 | Present assessments of risk of bias due to missing results (arising from reporting biases) for each synthesis assessed. | no |  |
| Certainty of evidence | 22 | Present assessments of certainty (or confidence) in the body of evidence for each outcome assessed. | Page5 |  |
| **DISCUSSION** | | | |  |
| Discussion | 23a | Provide a general interpretation of the results in the context of other evidence. | Page7-10 |  |
|  | 23b | Discuss any limitations of the evidence included in the review. | Page10 |  |
|  | 23c | Discuss any limitations of the review processes used. | Page10 |  |
|  | 23d | Discuss implications of the results for practice, policy, and future research. | Page10 |  |
| **OTHER INFORMATION** | | | |  |
| Registration and  protocol | 24a | Provide registration information for the review, including register name and registration number, or state that the review was not registered. | Page3 |  |
|  | 24b | Indicate where the review protocol can be accessed, or state that a protocol was not prepared. | Page3 |  |
|  | 24c | Describe and explain any amendments to information provided at registration or in the protocol. | Page3-4 |  |
| Support | 25 | Describe sources of financial or non-financial support for the review, and the role of the funders or sponsors in the review. | Page11 |  |
| Competing interests | 26 | Declare any competing interests of review authors. | Page11 |  |
| Availability of data,  code and other  materials | 27 | Report which of the following are publicly available and where they can be found: template data collection forms; data extracted from included studies; data used for all analyses; analytic code; any other materials used in the review. | Page11 |  |

Supplementary Materials 2

Database 1: PubMed

1. Stroke [MeSH Terms]

2. Brain Ischemia [MeSH Terms]

3. Intracranial Embolism and Thrombosis [MeSH Terms]

4. (stroke* or AIS or apople*) [Title/Abstract]

5. ((intracranial or carotid arter* or brain or cerebr*) and (isch*emi* or embolism or thrombosis or obstruct* or occlus* or block* or infarct* or clot*)) [Title/Abstract]

6. (cerebrovascular or cerebral arter*) and (accident or event* or disorder* or disease*) [Title/Abstract]

7. (anterior circulation or ACA) [Title/Abstract]

8. (large vessel occlusion* or large arter* occlusion* or LVO) [Title/Abstract]

9. or 1-8

10. Thrombectomy [MeSH Terms]

11. Embolectomy [MeSH Terms]

12. (mechanical or endovascular) and (thromb* or embol*)

13. (thrombectomy or embolectomy or EVT or MT) [Title/Abstract]

14. (bridg* or endovascular) and (therapy or treatment or intervention) [Title/Abstract]

15. ((clot* or thromb* or embol* or stent*) and (retriev* or disruption* or fragmentation)) [Title/Abstract]

16. (stent-retriever or aspiration or solitaire or trevo or preset or catch) [Title/Abstract]

17. or 10-16

18. (advanced imaging or perfusion imaging or advanced neuroimaging or perfusion or diffusion) [Title/Abstract]

19. (non-enhanced or noncontrast or non-contrast or nonen-hanced) [Title/Abstract]

20. (computed tomography or CT) [Title/Abstract]

21. (computed tomography perfusion or computed tomographic perfusion or CT perfusion or CTP or CT angiography or CTA) [Title/Abstract]

22. (magnetic resonance imaging or magnetic resonance imaging or perfusion magnetic resonance imaging or PMR or perfusion-weighted MRI or perfusion MRI or perfusion MR or MR perfusion or perfusion-weighted magnetic resonance or perfusion-weighted imaging or PWI or diffusion nuclear magnetic resonance or diffusion-weighted magnetic resonance imaging or diffusion magnetic resonance or diffusion-weighted MRI or diffusion MRI or diffusion weighted imaging or DWI or DWI-MRI or DWI-FLAIR mismatch) [Title/Abstract]

23. or 18-22

24. 9 and 17 and 23

Database 2: Embase

1. exp brain infarction/

2. exp brain ischemia/

3. exp cerebrovascular accident/

4. exp occlusive cerebrovascular disease/

5. (stroke* or AIS or apople*): ti, ab

6. ((intracranial or carotid arter* or brain or cerebr*) and (isch*emi* or embolism or thrombosis or obstruct* or occlus* or block* or infarct* or clot*)): ti, ab

7. (cerebrovascular or cerebral arter*) and (accident or event* or disorder* or disease*): ti, ab

8. (large vessel occlusion* or large arter* occlusion* or anterior circulation occlusion* or LVO): ti, ab

9. or 1-8

10. exp thrombectomy/

11. exp embolectomy/

12. (thrombectomy or embolectomy): ti, ab

13. (mechanical or endovascular) and (thromb* or embol*): ti, ab

14. (bridg* or endovascular) and (therapy or treatment or intervention): ti, ab

15. (stent-retriever or aspiration or solitaire or trevo or preset or catch): ti, ab

16. or 10-15

17. (advanced imaging or perfusion imaging or advanced neuroimaging): ti, ab

18. (non-enhanced or noncontrast or non-contrast or nonen-hanced) : ti, ab

19. (computed tomography or CT) : ti, ab

20. (computed tomography perfusion or computed tomographic perfusion or CT perfusion or CTP or CT angiography or CTA): ti, ab

21. (magnetic resonance imaging or perfusion magnetic resonance imaging or PMR or perfusion-weighted MRI or perfusion MRI or perfusion MR or MR perfusion or perfusion-weighted magnetic resonance or perfusion-weighted imaging or PWI or diffusion nuclear magnetic resonance or diffusion-weighted magnetic resonance imaging or diffusion magnetic resonance or diffusion-weighted MRI or diffusion MRI or diffusion weighted imaging or DWI or DWI-MRI or DWI-FLAIR mismatch): ti, ab

22. or 17-21

23. 9 and 16 and 22

Database 3: Cochrane Central Register of Controlled Trials

1. (stroke or large vessel occlusion or anterior circulation): ti, ab, kw

2. (thrombectomy or endovascular): ti, ab, kw

3. (perfusion or diffusion non-enhanced or noncontrast or non-contrast or nonen-hanced or computed tomography or magnetic resonance imaging): ti, ab, kw

4. 1 and 2 and 3

Supplementary Materials 3

To avoid heterogeneity, excluded articles are summarized

| Study ID | Country | Study design | Sample size | | Mean age (years) | | Symptom onset | Treatment | Outcomes | Variables adjusted for |
| --- | --- | --- | --- | --- | --- | --- | --- | --- | --- | --- |
|  |  |  | CTP or MRI | NCCT/CTA | CTP or MRI | NCCT/CTA |  |  |  |  |
| Chalouhi 2013 | America | P | 94 | 38 | 66.5 ± 1.6 | 63.5 ± 2.13 | <8h | MT | ①②③④ | NR |
| Sheth 2013 | America | P | 270 | 286 | 66±15 | 65±15 | <8h | EVT | ②③ | age, NIHSS, clot location,et al. |
| Prabhakaran 2014 | Multicenter | P | 76 | 138 | 72.4 (14.1) | 64.4 (16.3) | <8h | EVT | ②③⑤ | age, calendar year, initial NIHSS score, et al. |
| Vagal 2016 | Multicenter | P | 61 | 369 | 69 (59–75) | 68 (57–76) | NR | EVT | ② | age, NIHSS strata, et al. |
| Bouslama 2017 | America | R | 212 | 212 | 63.92±15.23 | 67.83±14.55 | NR | EVT | ②③⑤ | NR |
| Koneru 2018 | NR | R | 164 | 85 | 70 | 72 | NR | MT | ②⑤ | NR |
| Kim 2019 | Korea | P | 986 | 279 | 69.2 (11.4) | 68.9 (12.6) | <6h, >6h | EVT | ①②③④⑤ | age, male gender, baseline NIHSS score, et al. |
| Provost 2019 | America | P | 299 | 102 | 67 (54–74) | 65 (54–74) | <5h | MT | ②③⑤⑥ | NR |
| Kim 2020 | Korea | R | 33 | 34 | 67.0±11.5 | 71.1±10.9 | >4.5h | MT | ②⑤ | NR |
| Lin 2020 | China | R | NR | 124/75 | NR | 62.56(11.83) | NR | MT | ③ | NR |
| Ren 2021 | China | P | 418 | 476 | 67 (59–75) | 65 (56–74) | <6h | MT | ①②③④⑤ | age, male gender, baseline NIHSS score, et al. |
| Dekker 2021 | Netherlands | P | 85 | 21 | 67.4 (14.7) | 65.8 (14.4) | <6.5h,>6.5h | EVT | ②③④⑤ | NR |
| Fischer 2022 | Swiss | P | 3741 | 7308 | 69.0±15.2 | 72.1±14.4 | <24h | MT | ②③④ | NR |
| Krebs 2022 | Austrian | P | 2,226 | 14,573 | 72 (62–81) | 75 (65–83) | NR | MT | ①②④ | age, sex, baseline NIHSS, et al. |
| Garcia-E 2023 | NR | R | 52 | 79 | 65 [58–74] | 64 [52–70] | NR | MT | ②③ | NR |

P: Prospectively R:retrospective MT: mechanical thrombectomy EVT: endovascular therapy NR:no report

Outcome:①mRS 0-1; ②mRS 0-2; ③Mortality; ④sICH; ⑤TICI 2b-3; ⑥SH: Symptomatic hemorrhage;

Supplementary Materials 4

Quality assessment of included studies with the Newcastle–Ottawa Scale

| Study name | Selection | Comparability | Outcome | Overall score |
| --- | --- | --- | --- | --- |
| Leslie-M 2016 | *** | ** | *** | 8/9 |
| Sarraj 2018 | *** | ** | *** | 8/9 |
| Desai 2019 | **** | ** | *** | 9/9 |
| Nogueira 2021 | *** | ** | ** | 7/9 |
| Ren 2021 | *** | ** | *** | 8/9 |
| Herzberg 2021 | **** | ** | *** | 9/9 |
| Dhillon 2022 | *** | ** | *** | 8/9 |
| Porto 2022 | *** | ** | ** | 7/9 |
| Jadhav 2022 | *** | ** | *** | 8/9 |
| Nguyen 2022 | ** | ** | *** | 7/9 |
| Almekhlafi 2022 | *** | ** | ** | 7/9 |
| Cheng 2023 | *** | ** | *** | 8/9 |
| Miao 2023 | **** | ** | *** | 9/9 |
| Sakakibara 2023 | *** | ** | *** | 8/9 |

Note: A study can be awarded a maximum of one star for each numbered item within the Selection and Outcome categories. A maximum of two stars can be given for Comparability.

Selection

1) Representativeness of the exposed cohort

a) truly representative of the average (describe) in the community*

b) somewhat representative of the average in the community*

c) selected group of users eg nurses, volunteers

d) no description of the derivation of the cohort

2) Selection of the non-exposed cohort

a) drawn from the same community as the exposed cohort *

b) drawn from a different source

c) no description of the derivation of the non-exposed cohort

3) Ascertainment of exposure

a) secure record (eg surgical records) *

b) structured interview *

c) written self-report

d) no description

4) Demonstration that outcome of interest was not present at start of study

a) yes *

b) nი

Comparability

1) Comparability of cohorts on the basis of the design or analysis

a) study controls for(select the most important factor) *

b) study controls for any additional factor (This criteria could be modified to indicate specific control for a second important factor.) *

Outcome

1) Assessment of outcome

a) independent blind assessment*

b) record linkage*

c) self-report

d) no description

2) Was follow-up long enough for outcomes to occur

a) yes (select an adequate follow up period for outcome of interest) *

b) no

3) Adequacy of follow up of cohorts

a) complete follow up-all subjects accounted for *

b) subjects lost to follow up unlikely to introduce bias-small number lost-> 60 % (select anadequate %) follow up, or description provided of those lost) *

c) follow up rate< % (select an adequate %) and no description of those lost

d) no statement

Supplementary Material 5 Funnel plots for impact of stroke imaging selection modality on endovascular thrombectomy outcomes in the early and extended time windows.( a: functional independence;b: sICH; c: successful reperfusion;d: mortality.)

a
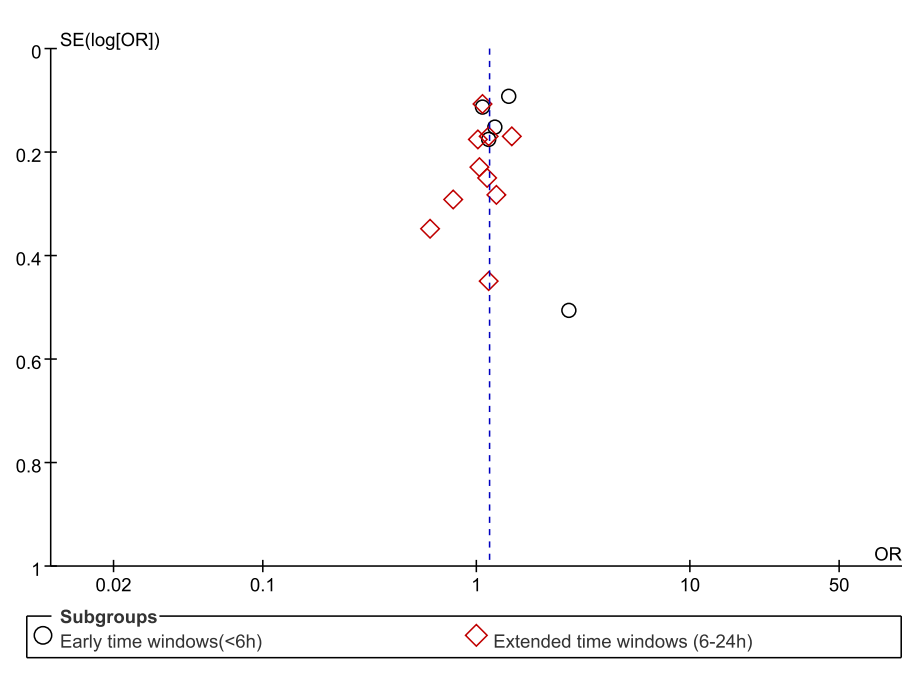
b
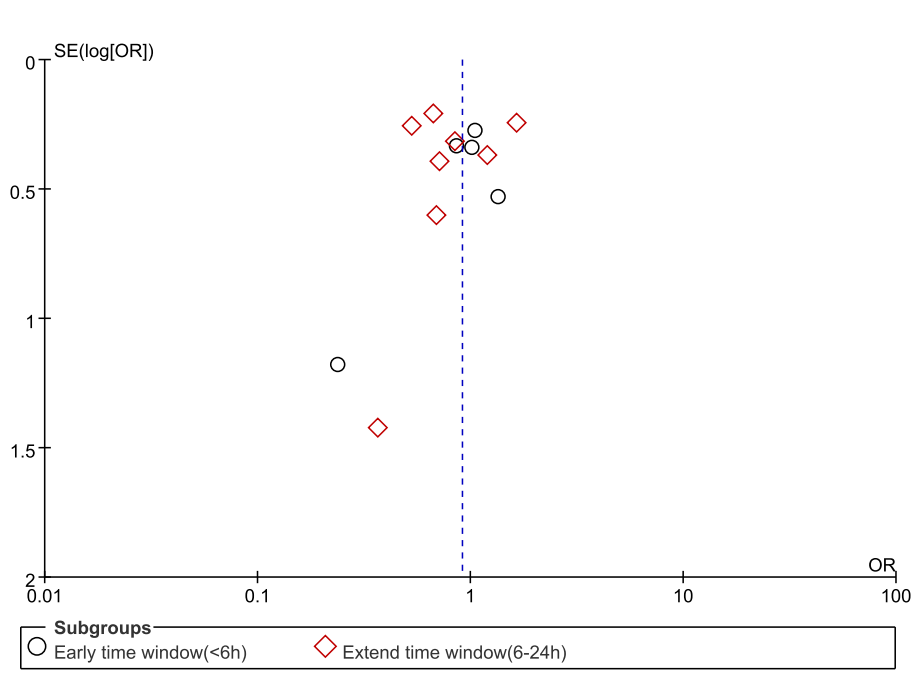


c
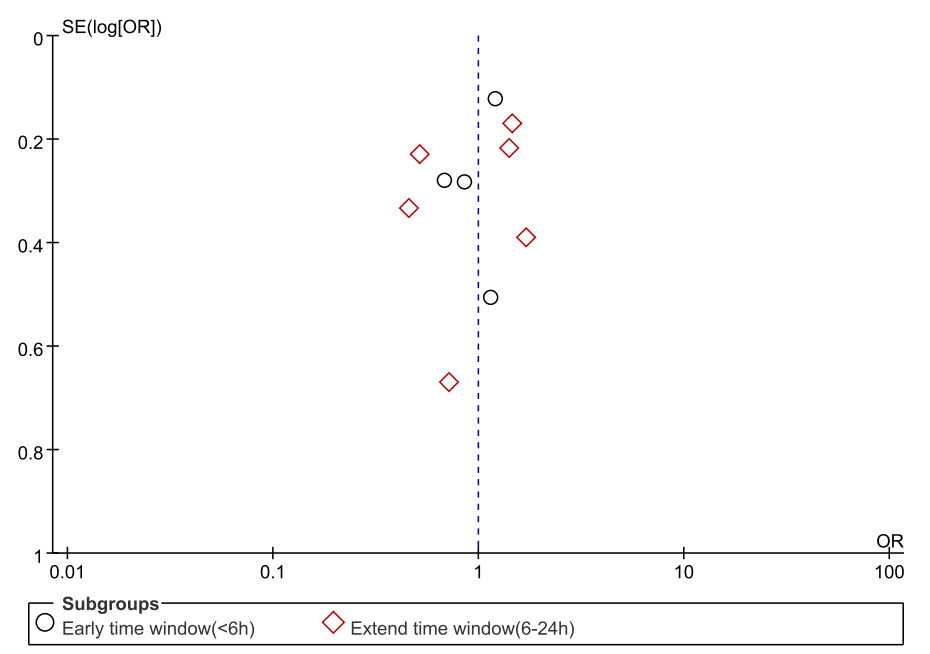
d
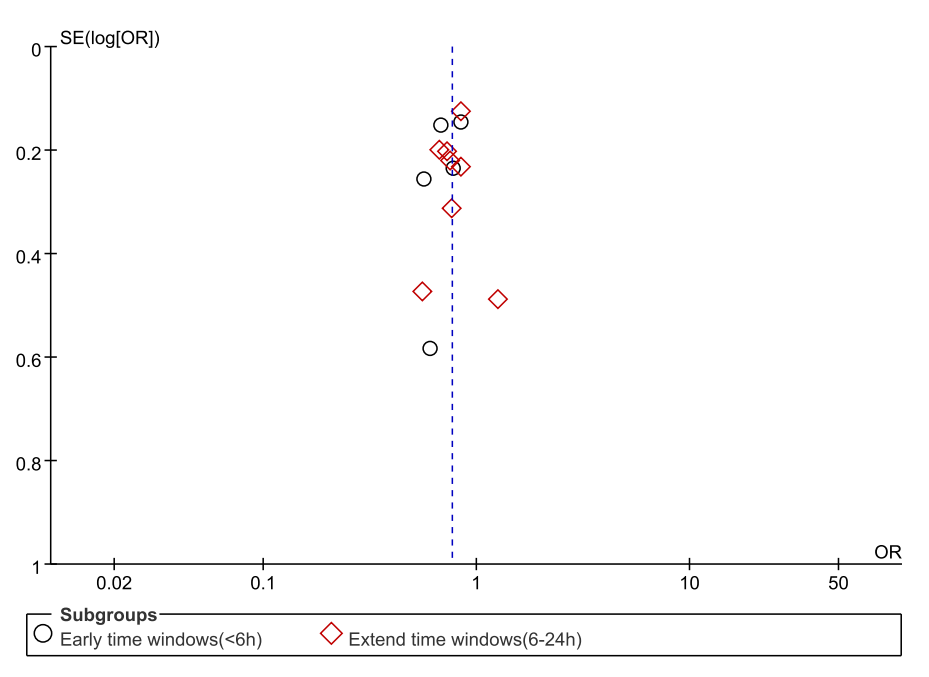

Supplement: Supplementary file 1 — Supplementary Materials [file BRB3-14-e3530-s001.docx]
